# Conservative vs. Surgical Management of Condylar Fractures in Pediatric Populations: Complications and Factors for Consideration

**DOI:** 10.3390/children12030323

**Published:** 2025-03-03

**Authors:** Chaim Ohayon, Amit Perelman, Adi Katz Biton, Andrei Krasovsky, Nidal Zeineh, Jiriys George Ginini, Adi Rachmiel, Amir Bilder, Omri Emodi

**Affiliations:** 1 Oral and Maxillofacial Surgery Department, Rambam Health Care Campus, Haifa 3109601, Israel; 2 Department of Medicine, Technion Israel Institute of Technology, Haifa 3109601, Israel

**Keywords:** mandibular condyle/injuries, mandibular fractures, facial trauma/complications, pediatrics, temporomandibular joint/growth and development, maxillomandibular fixation/methods, fracture fixation internal/methods

## Abstract

Background: The optimal treatment decision for pediatric condylar fractures is influenced by various factors, including the child’s age, fracture type, degree of displacement, and the presence of concomitant injuries. While non-surgical treatments are generally preferred due to high remodeling capacity in children, there remains a lack of comprehensive research comparing the long-term outcomes of open reduction internal fixation (ORIF) versus conservative management. Methods: Retrospective analysis of medical records of 71 pediatric patients (aged 0–18 years) treated for condylar fractures at the Department of Oral and Maxillofacial Surgery, Rambam Healthcare Campus, between 2010 and 2020 was completed. Gender, age, admission date, cause of injury, treatment methods, length of hospital stay, follow-up duration, and follow-up status were studied to help determine association with modality of treatment and complications. Results: No statistically significant differences were seen in complication rates across different age groups, genders, trauma etiologies, fracture sites (head/neck/base), laterality of fractures, nor was there an impact on choice of surgical vs. conservative intervention. Similar length of hospital stay was observed, even in cases with delayed surgical intervention. There was also no statistical significance of injury distribution based on socioeconomic standing. Conclusions: Our research showed similar rates of complications in both surgically treated and conservatively treated cases. This solidifies the importance of practitioner experience, as well as comprehensive anamnesis to help caretakers most effectively determine the optimal treatment for each patient. As the surgical study group is substantially smaller than the conservatively treated group, large-scale prospective studies with extended follow-up will more conclusively help solidify results and establish guidelines.

## 1. Introduction

Facial fractures in pediatric patients, although less common than in adults, pose significant clinical challenges due to their potential impact on injury severity, length of hospital stay, morbidity, and mortality [1]. Craniofacial fractures account for less than 5% of pediatric hospitalized trauma cases. However, facial fractures are associated with an increased incidence of severe brain injuries compared to those without such fractures. The prevalence and types of facial fractures vary across different age groups. The most commonly observed fractures in this population, in descending order of frequency, are mandibular, nasal, and maxillary/zygoma fractures, often resulting from motor vehicle collisions, violence, and falls [2]. Mandibular fractures occur more frequently in older children, while nasal and maxillary fractures are more common in toddlers, influenced by the relative strength of the mandible and differing mechanisms of injury [2].

The mandibular condyle is crucial in mandibular growth and development, undergoing various developmental stages from infancy to late adolescence. The anatomical changes during these stages influence the patterns of mandibular condylar fractures [3,4,5]. Mandibular fractures often involve the condyle, even more frequently in children than in adults, and are more common in boys than in girls. Some studies maintain that younger children tend to experience intracapsular and high neck fracture, whereas other studies maintain that most are extracapsular fractures in children [4]. 

Mandibular condylar fractures in children can lead to a range of complications, including malocclusion, masticatory dysfunction, facial asymmetry, restricted mandibular movements, and temporomandibular joint disorders. Timely recognition of these fractures, appropriate treatment decisions, and proper follow-up are essential to minimizing complications and restoring jaw function and symmetry [6]. Treatment approaches for mandibular condyle fractures include open reduction with internal fixation (ORIF) and conservative non-invasive methods such as soft diet with potential maxillomandibular fixation (MMF) or orthodontic treatment. It should be noted that due to the growing mandible, ORIF frequently necessitates removal of plates within a six-to-eight-month period in growing children, except when absorbable plates are used. The optimal treatment for pediatric condylar fractures may be influenced by the child’s age, fracture type, degree of displacement, and presence of concomitant injuries [7,8,9]. 

Despite a general preference for non-surgical management due to the children’s remodeling capabilities, there remains a lack of comprehensive research on the long-term outcomes of ORIF compared to closed treatment. In accordance with accepted treatment protocol, our center also treated the majority of pediatric condylar fractures using conservative treatment. Though limited due to significant discrepancy in group sizes, this retrospective study aims to analyze the outcomes and complications associated with various treatment methods for pediatric condylar and subcondylar fractures admitted to Rambam Health Care Campus between 2010–2020 to help give insight for the selection of the treatment modality in such fractures among children. 

## 2. Materials and Methods

### 2.1. Data Collection

The entire study was based on STROBE recommendations for retrospective analysis (Appendix A). We gathered data from medical records of 75 pediatric patients treated at Rambam ER for condylar fractures between 2010 and 2020. We also collected data from the Israel Central Bureau of Statistics. Data regarding gender, birthdate, age, socioeconomic status, admission date details (day, month, weekday type—holiday, weekend, or workday), cause of injury, summary of all facial fractures, treatment modality, length of hospital stay, follow-up duration, current follow-up status (on-going follow-up/quit from follow-up/finished follow-up), complications, and imaging details were collected and analyzed. Details of treatment, whether conservative or surgical, were gathered to compare final results. Treatment modality selected was based on age, compliance, and degree of displacement with a general tendency towards conservative treatment when functionally possible. Following injury, patients were initially accepted and hospitalized for monitoring. After their discharge, they remained under close follow-up (weekly), and as function improved, time between appointments was gradually lengthened. Each case was presented, and the ultimate choice of treatment modality was determined by a consilium of surgeons from our center. Follow-up was also not with a predetermined or specific doctor. This approach was utilized in all cases at the department in order to minimize individual practitioner bias and preference.

### 2.2. Classification

**Figure 1:**
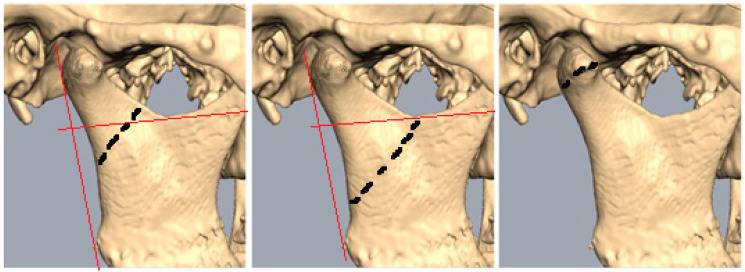
Line drawn perpendicular to the posterior ramus line via sigmoid notch marks the cutoff point. Black dotten lines mark level of fracture. Condylar neck: more than 50% above line; subcondylar: 50% below line; condylar head: fracture line through condylar head.

The cause of injury was further divided into five groups: falls, violence, motor vehicle accidents, sport-related accidents, and two-wheeled vehicle accidents. Surgical treatment options included ORIF, or ORIF with MMF. Conservative management included observation, soft diet, physiotherapy, and possible maxillomandibular fixation. Imaging modalities used included CT, panoramic, and plane X-ray radiographs (including Town’s, AP, cephalometric, etc.). Ongoing orthodontic treatment sometimes used in these types of injuries was not included in this study. Subsequently, each imaging category was divided into left or right sides and then into mandibular condyle head/base/neck fractures based on the Strasbourg Osteosynthesis Research Group method (Figure 1). Specific details were recorded for condylar head fractures, noting any instances of displacement and specifying whether it was lateral or medial pole displacement. For neck and subcondylar fractures, displacement angle was measured and recorded. Additionally, complications of injury were divided into distinct categories: none, asymmetry including mandibular deviation, open bite, limited mouth-opening capacity, neurologic injury, mastication or functional disturbances, auscultation noises in TMJ, and pain during palpation of TMJ.

### 2.3. Statistics

Descriptive statistics: means, standard deviations, medians, percentiles, and ranges were calculated to all included variables. Differences in continuous variables among the groups with complications and without complications were analyzed using a two-tailed *t*-test or Mann–Whitney U test. Differences in categorical variables were determined using a Pearson chi-square or Fisher’s exact test. Statistical analysis was performed using SPSS software, version 28. A two-tailed value of *p* ≤ 0.05 was considered significant. 

## 3. Results 

**Figure 2:**
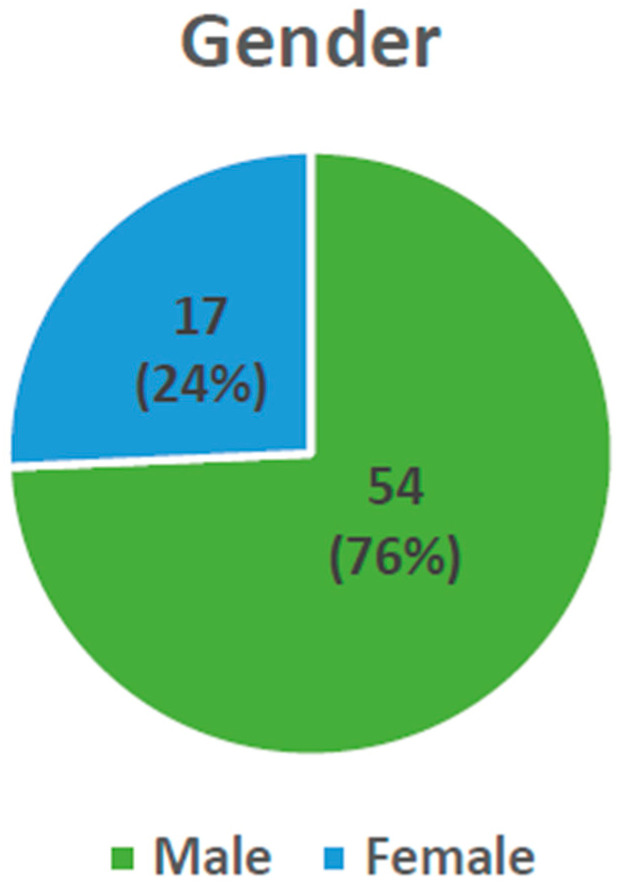
Distribution of injury based on gender.

Out of the 71 patients who met the inclusion criteria, 54 of them were male and 17 of them were female (Figure 2). There was no significant difference in gender ratio between the patients who were treated surgically and those who were treated conservatively. Among the men’s population, 37 patients did not have complications and 17 had complications, whereas among the women’s population there were 11 patients without complications and six with complications. The gender of the patient was found to be statistically insignificant in the likelihood of complications. 

**Figure 3:**
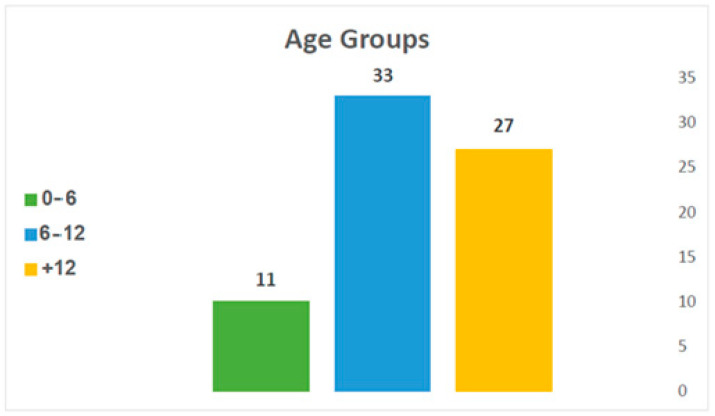
Distribution of injury by age.

The patients were divided into three age groups according to dentition development: ages 0–6 years old (not including 6, primary dentition), 6–12 years old (not including 12, mixed dentition), and 12 years old at least (permanent dentition) (Figure 3). Age was not a statistically significant factor when comparing conservative vs. surgical intervention. The mean age was 9.99 ± 4.7 in the group without complication, compared to 11.63 ± 4.44 in the group with complications. This difference was also non-significant.

**Figure 4:**
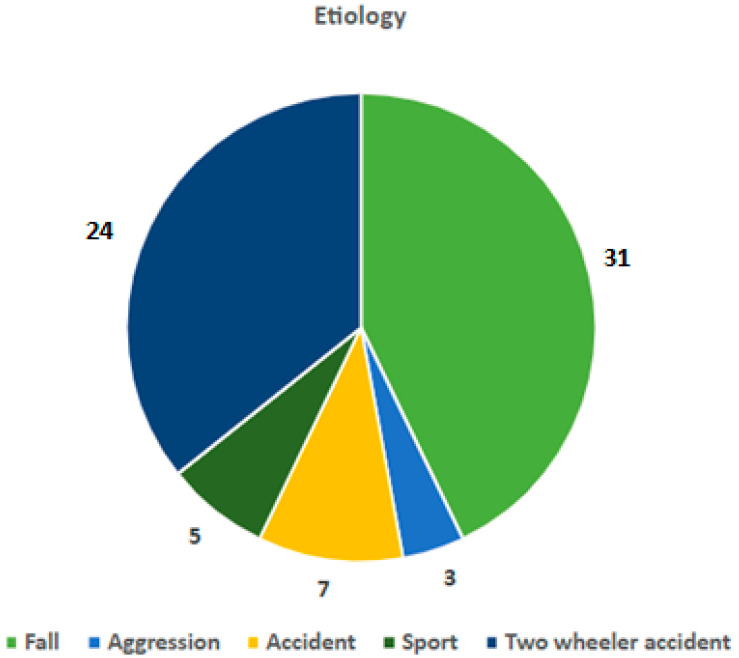
Distribution of injury by mechanism.

The trauma’s etiologies were classified into five main categories: falls, violence, motor vehicle accidents, sport injuries, and two-wheeled vehicle accidents. Each patient was attributed to one of the categories (Figure 4). No statistically significant difference was found between the surgical vs. conservative treatment cases in each of these categories. In other words, choice of treatment was not influenced by etiology of the fracture. For 32 patients, a fall was the cause of trauma: 24 of them were without any complication, and eight patients had at least one complication. For patients for whom violence was the cause of the trauma, two patients were without complication and one patient had a complication. In cases where motor vehicle accidents caused the trauma, three patients were without complication and four patients experienced complications. Sports injuries led to four patients with no complications and one patient with a complication. Those suffering injuries from two-wheeled vehicle involvement, 15 patients were without any complication and nine had complications. There was no statistical significance for the etiology of injury and the likelihood to experience complications.

**Figure 5:**
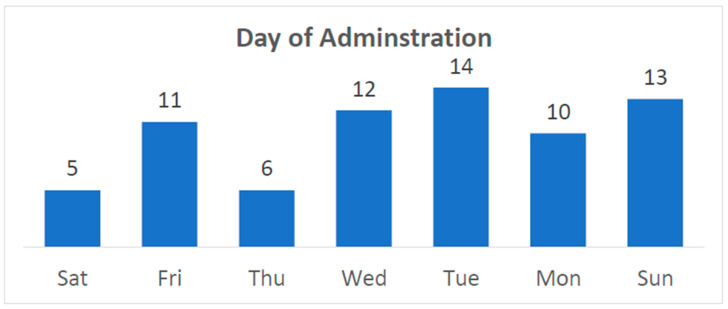
Day of the week of admission.

The month, day of week (Figure 5), and type of day (weekday, holiday, or weekend) when admitted to the hospital was also statistically insignificant in terms of complications.

In total, the patients had 77 condylar fractures (classified by Loukota et al. [10,11] classification system, Figure 1): 35 were head fractures (eight of which were bilateral), seven were neck fractures (three of which were bilateral), and 35 were subcondylar fractures (three of which were bilateral).

There were 26 patients who had at least one more facial fracture besides the condylar fractures, including 24 patients who had an additional mandibular fracture. Of the 45 patients with sole condylar fracture: 23 of them had unilateral subcondylar fracture, one had bilateral condylar neck fracture, 18 of the patients had unilateral head fractures, and three had bilateral head fractures. 

Occurrence of fractures at different levels were compared (Table 1). Our findings indicate that the type of fracture does not influence the likelihood of complications, as there were no significant differences in the frequency of head, neck, and subcondylar fractures between patients who experienced complications and those who did not. 

There was no significant statistical difference in the rates of unilateral and bilateral condylar fractures between patients who experienced complications and those who did not, indicating that the laterality of condylar fracture was not a significant factor in the development of complications. The number of days that passed between admission until undergoing surgery were 2.29 ± 1.6 days in the group of patients who had complications and 1.47 ± 0.90 days in the group of patients who did not. This difference was also statistically nonsignificant. 

Days of hospitalization were also nonsignificant between the group with complications (4.44 ± 2.58 days) and without complications (4.07 ± 1.62 days). 

We defined non-surgical treatment as supportive treatment including or not including MMF; 64 patients underwent such treatment. Surgical treatment that included ORIF ± MMF was the treatment modality in six cases. One patient refused treatment and left the hospital on his own. The determination to pursue either conservative or surgical treatment was reached following a consultation between the resident physician and the on-call attending. In older patients, or in cases where there was insufficient compliance to achieve optimal results with conservative treatment, as well as in cases of significant displacement or cases of concomitant injuries, the tendency was to opt for surgical treatment for improved outcomes.

A total of 43 of the patients treated conservatively did not suffer complications, whereas 21 did experience complications. Of those treated surgically, two did experience complications. There was no statistical significance in likelihood of complication between surgery vs. non surgery approach.

During the follow-up period, which varied among patients, 24 patients suffered complications (Table 2). A total of 16 patients reported asymmetry (which included deviation in mouth opening), six had limited mouth opening of less than 35 mm, and one patient had nerve damage.

The analysis revealed that the differences in the length of follow-up periods were not statistically significant. The probability of a patient having a complication was not influenced by the duration of the follow-up period. It was also determined that the complications were not influenced by the follow-up status of the patients, whether they had completed follow-up, were still undergoing follow-up, or had discontinued.

## 4. Discussion 

The mandibular condylar region has three developmental stages divided by ages: 0–2 years, 3–12 years, and 12–18 years [5]. In infancy, the glenoid fossa is shallow, and as one grows, the condylar process and glenoid fossa start to develop, but still there is great potential of regenerating and remodeling. In late adolescence, the mandibular condylar region has the anatomic pattern as in an adult. Osteogenesis still exists, but the condylar remodeling ability diminishes dramatically [5].

Indications for open surgery in pediatric mandibular condylar fractures is a debated topic, with ongoing efforts to achieve consensus on the matter. Among pediatric condylar process fractures, fractures with a fragment that is totally displaced from the glenoid fossa are frequent [12]. Such fractures require more extensive remodeling to maintain TMJ function and prevent future mandibular asymmetry, incomplete remodeling of the condylar process, and TMJ dysfunction [3]. Some of the proposed indications to choose open treatment in children focus primarily on severe mandibular injuries and severe displacement that limits the function of the TMJ [7]. Other contraindications for MMF include lack of patient compliance with soft diet and physical therapy, as well as problematic anatomy of primary teeth that renders MMF relatively unstable. TMJ ankylosis in children is another risk of closed treatment for extended periods of time [12,13].

Deleyiannis et al., 2006 focused on the long-term outcomes of treating pediatric displaced mandibular condylar fractures with ORIF, the radiologic and TMJ outcomes were like that of nonsurgical treatment. The potential advantage of ORIF should be weighed against the surgical risks and the logistic challenge of frequent long-term follow-up, which is particularly important because of the possible disruption of mandibular growth. The study concluded that despite successful potential of ORIF, nonsurgical management should be the primary treatment option for displaced pediatric condylar fractures as the advantages of ORIF do not consistently outweigh IMF [14].

In contrast, a study of displaced mandibular condylar fractures of over 45 degrees maintained the importance of open treatment and joint visualization. Attention is called to the articular disc within the TMJ maintaining that a damaged disc in mandibular condylar fractures may lead to painful clicking and even crepitations within the TMJ. It concluded that in significantly displaced fractures (deviation of 30 degrees or less were considered mild) ORIF was the treatment of choice, helping fix the disc to its original position and maintain contact with the condyle [7].

We were unable to compare treatment types (surgical vs. conservative) across age groups due to a small sample size in each category, which did not provide enough statistical power. Furthermore, a comparison of the disparities in complications stemming from reliance on surgery was not feasible between the age groups due to insufficient available data. None of the variables studied (gender, mechanism and severity of trauma, timing of admission, age group, fracture site, laterality of mandibular condylar fractures, and the presence of surgery or other interventions) significantly impacted the rate of complications. 

An additional factor to consider is the socioeconomic status of the patients. Unlike the distribution of the population, which follows a standard bell curve, the injuries appear to occur more frequently within the lower range of the spectrum. This may reflect a higher predilection of such mandibular condylar injuries among lower-income families. The data for this study was tested to determine a correlation between socioeconomic level and the likelihood of injury, and although no statistical significance was found, there may be room for larger test groups. Furthermore, the cause of such injuries may vary based on socioeconomic status.

## 5. Conclusions

Treatment of mandibular condylar fractures in children remains a debated topic in the literature, with various approaches discussed including conservative treatments and more aggressive ORIF treatments. More recent approaches try and minimize risks of traditional ORIF, which improve the end result by employing endoscopic approaches [15]. While promising, there is still no wide consensus to the best treatment modality among pediatric patients. Although common injuries, pediatric condylar fractures present a unique set of challenges. Treatment modalities used in adult cases cannot necessarily be adapted for the younger population due to the potential ramifications of these treatments on the inherent growth potential of the mandible [16].

Through a retrospective analysis of pediatric condylar fractures treated at Rambam, we attempted to find significant differences between the two study groups. None of the variables examined in the study were found to significantly affect the complication rate when choosing between conservative and surgical treatment of mandibular condylar fractures. Surgery has its inherent risks. As such, unless there is a clear indication for surgical intervention, we recommend continuing to choose the conservative approach. Additional supplemental treatments can also be considered for inclusion in treatment, such as orthodontics. However, that was not included in the scope of this study. Larger-scale studies, or even prospective studies with long-term follow-up periods, potentially involving multiple centers, may help determine a definitive treatment guideline with significant findings to sharpen a conclusive protocol for treatment of pediatric mandibular condylar fractures. 

## Figures and Tables

**Table 1:** Occurrence of fractures at different levels.

	Cases	Age	Left	Right	Bilat	Surgery	Complications	Additional Fracture
Condylar Head	35	9.2	15	12	8	10	12	12
Condylar Neck	7	7.6	3	1	3	4	1	6
Subcondylar	35	12	19	13	3	15	11	11

**Table 2:** Occurrence of complications based in surgical vs. conservative treatment.

	Complication	No Complication	Marginal Row Totals
Surgery	2	4	6
No surgery	21	43	64
Marginal column totals	23	47	70 (grand total)

## Data Availability

The raw data supporting the conclusions of this article will be made available by the authors on request.

## Appendix A. Based on the Official STROBE Checklist [17]

	**Item No**	**Recommendation**	
Title and Abstract	1	(a) Study Design	✓
		(b) Informed summary	✓
Introduction			
Background	2	Explain rationale	✓
Objectives	3	Specific objectives, hypotheses	✓
Methods			
Study Design	4	Key elements	✓
Setting	5	Setting, location, dates, recruitment, follow-up	✓
Participants	6	(a) Eligibility	✓
		(b) Matching criteria	✓
Variable	7	Clearly defined outcomes, exposures, predictors, confounders, modifiers	✓
Sources/measurement	8	Methods of assessment, comparability	✓
Bias	9	Efforts to address	✓
Study size	10	Explanation	✓
Quantitative variables	11	Group selection	✓
Statistical methods	12	(a) Describe, including confounding control	✓
		(b) Subgroup interactions	✓
		(c) Missing data addressed	✓
		(d) Loss to follow-up address	Results section
		(e) Sensitivity analysis	Results section
Results			
Participants	13	(a) Breakdown at each stage	✓
		(b) Reasons for non-participation	✓
		(c) Flow design	n/a
Descriptive data	14	(a) Demographic, clinical, social characteristics of participants	✓
		(b) Number with missing data	Not included
		(c) Average follow-up time	✓
Outcome data	15	Outcome events, summary	✓
Main results	16	(a) Raw data and adjustments for confounders	Described
		(b) Category boundaries in cases of multiple variables	✓
		(c) Relative vs. absolute risk over time	n/a
Other analyses	17	Description of all subgroup and interaction analyses	✓
Discussion			
Key results	18	Summarize key points, reference objectives	✓
Limitations	19	Description, potential bias, imprecision	✓
Interpretation	20	Overview when accounting for limitation and literature of similar studies	✓
Generalisability	21	External validity of study results	Conclusion
Other information			
Funding	22	If relevant	n/a
